# Enantiomeric Resolution and Absolute Configuration of a Chiral δ-Lactam, Useful Intermediate for the Synthesis of Bioactive Compounds

**DOI:** 10.3390/molecules25246023

**Published:** 2020-12-19

**Authors:** Roberta Listro, Giacomo Rossino, Serena Della Volpe, Rita Stabile, Massimo Boiocchi, Lorenzo Malavasi, Daniela Rossi, Simona Collina

**Affiliations:** 1 Department of Drug Sciences, University of Pavia, via Taramelli 12, 27100 Pavia, Italy; roberta.listro01@universitadipavia.it (R.L.); giacomo.rossino01@universitadipavia.it (G.R.); serena.dellavolpe01@universitadipavia.it (S.D.V.); rita.stabile01@universitadipavia.it (R.S.); simona.collina@unipv.it (S.C.); 2 Centro Grandi Strumenti, University of Pavia, via Bassi 21, 27100 Pavia, Italy; massimo.boiocchi@unipv.it; 3 Department of Chemistry, University of Pavia, via Taramelli 12, 27100 Pavia, Italy; lorenzo.malavasi@unipv.it

**Keywords:** lactam scaffold, enantioselective HPLC, chiral resolution, X-ray diffraction, absolute configuration assignment

## Abstract

During the past several years, the frequency of discovery of new molecular entities based on γ- or δ-lactam scaffolds has increased continuously. Most of them are characterized by the presence of at least one chiral center. Herein, we present the preparation, isolation and the absolute configuration assignment of enantiomeric 2-(4-bromophenyl)-1-isobutyl-6-oxopiperidin-3-carboxylic acid (*trans*-**1**). For the preparation of racemic *trans*-**1**, the Castagnoli-Cushman reaction was employed. (Semi)-preparative enantioselective HPLC allowed to obtain enantiomerically pure *trans*-**1** whose absolute configuration was assigned by X-ray diffractometry. Compound (+)-(*2R,3R*)-**1** represents a reference compound for the configurational study of structurally related lactams.

## 1. Introduction

Drug discovery is a complex process aimed at identifying new biologically active compounds with a high degree of structural novelty [1]. It can be driven by the exploration of the chemical space around a drug or a selected scaffold, the core structure of the molecular framework. Scaffolds are used as starting points for compound synthesis or diversification and, therefore, their study represents an effective and promising approach for finding new potent drugs [2,3,4,5].

**Figure 1:**
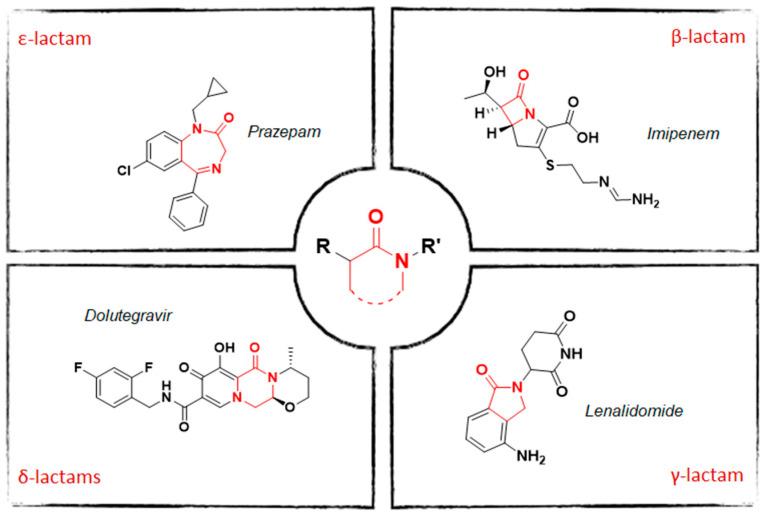
Examples of marketed drugs based on the lactam scaffold.

In recent years, substituted lactams have emerged as an important pharmacophore for several drug classes, with a large spectrum of biological outcomes and various therapeutic applications [6,7,8]. From a structural standpoint, they can be four-, five-, six- and seven-membered rings, called β-lactams, γ-lactam, δ-lactam and ε-lactam, respectively. Several drugs with a lactam structure have already reached the market, and a few representative examples are reported in Figure 1. These include antibiotics with a β-lactam structure (i.e., penicillins, cephalosporins, monobactams, carbapenems) [9], the γ-lactam *Lenalidomide* (a derivative of *Thalidomide* endowed with immunomodulatory and antiangiogenic activity) [10,11], the δ-lactam *Dolutegravir* (a HIV-1 integrase inhibitor) [12] and benzodiazepines based on the ε-lactam scaffold (i.e., *Prazepam*), well known allosteric modulators of the receptor GABA A. [13].

**Figure 2:**
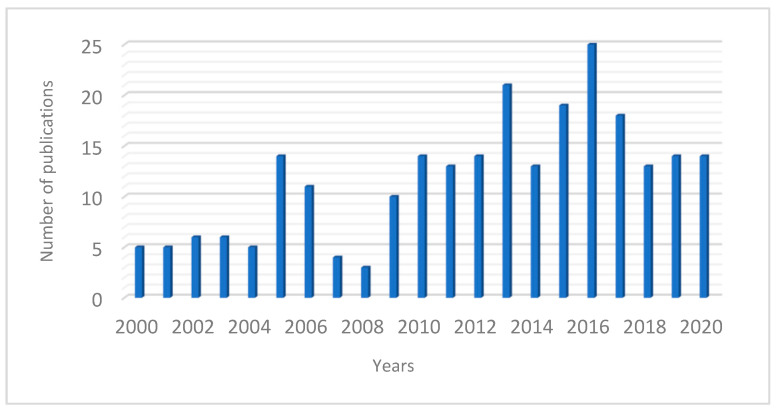
Papers published per year. Source Scopus (31 October 2020), key words “lactams” and “medicinal chemistry”.

In the last few years, the interest of MedChem researchers towards lactams as chemical scaffolds has grown, as evidenced by the increasing number of publications (Figure 2), due to their high potential for discovering drug candidates. In fact, lactams possess attractive features such as versatility and easy derivatization, and, additionally, they are conformationally restricted scaffolds with peptidomimetic features. Thus, they can be used to improve the potency, selectivity, and metabolic stability of peptide-based drugs [7].

Several MedChem groups have focused on the synthesis of new molecular entities based on γ- or δ-lactam scaffolds as highly versatile key intermediates, leading to the discovery of new anti-trypanosomal, anti-biofilm and anti-inflammation agents [14,15,16,17,18]. For instance, Delong et al. studied novel α-methylene-γ-lactams, α-arylidene-γ and δ-lactams, with novel antifungal properties against *Colletotrichum orbiculare* [19], whereas Davoren et al. prepared a series of molecules with lactam scaffolds and identified a number of γ- and δ-lactams able to function as positive allosteric modulators (PAMs) of muscarinic receptors (M1) [20]. Lastly, by applying a combined molecular modelling-STD NMR approach, our group recently identified a promising ligand with a δ-lactam scaffold able to bind the RNA-binding protein HuR [21,22,23], which interacts with target mRNAs, leading to the formation of HuR–mRNA complexes involved in several physio-pathological conditions [24,25,26].

**Scheme 1:**
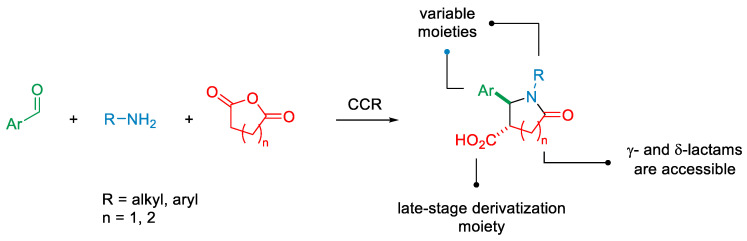
Scheme of the Castagnoli-Cushman reaction.

Given the high interest in lactams, extensive efforts have been directed towards the identification of versatile synthetic strategies for obtaining lactams. Among the different methodologies exploited so far, it is worth mentioning the Beckmann rearrangement, the Staudinger procedure and the Schmidt reaction as well as cascade, tandem and multi-component reactions [27,28]. From the perspective of efficiency, medicinal chemistry projects can advance faster with simple reactions that can use easily available reagents (e.g., amines, aldehydes and carboxylic acids) and that require low optimization procedures. Such reactions can be successfully used for preparing compound libraries with a high degree of molecular diversity, thus speeding up the drug discovery process. From this perspective, the Castagnoli-Cushman reaction (CCR) seems to be a robust procedure suitable for affording γ- or δ-lactams with two points of structural variability, plus one additional carboxylic portion offering a site for derivatization in one or more steps, and two stereogenic centers (Scheme 1). The CCR generally proceeds with a high degree of diastereoselectivity, yielding predominantly the trans-configured racemic mixture [29,30,31,32,33]. Recently, Ryabukhin et al. successfully applied the CCR for preparing a potential lead-oriented library based on a lactam scaffold. Specifically, starting from two different anhydrides, 44 aldehydes and 44 amines, and applying a one-pot parallel synthesis procedure, they prepared a series of 132 lactams with high yields [34].

The stereoselectivity of the CCR is a particularly appealing feature, considering that the lactams so far proposed as drug templates, or more generally drugs with a lactam structure, are mainly characterized by the presence of at least one chiral center [8,35,36]. This is not surprising, since it is well recognized that chiral small molecules play a relevant role in different areas of the study of biological systems (for example, drug discovery and chemical biology). Most processes in nature are inherently shaped by chirality and therefore, in the drug discovery process, chirality is often exploited to modulate the interaction between small and macromolecules [37,38,39,40]. Despite its significant role in the interaction with the target, small-molecule chirality has not always been investigated along the entire process of drug design and development.

Specifically, in the case of CCR-derived lactams, there has been poor interest on the enantiomeric resolution of these compounds via liquid chromatography. To the best of our knowledge, only Kronn et al. reported on the separation of a δ-lactam via supercritical fluid chromatography (SFC) [41].

**Figure 3:**
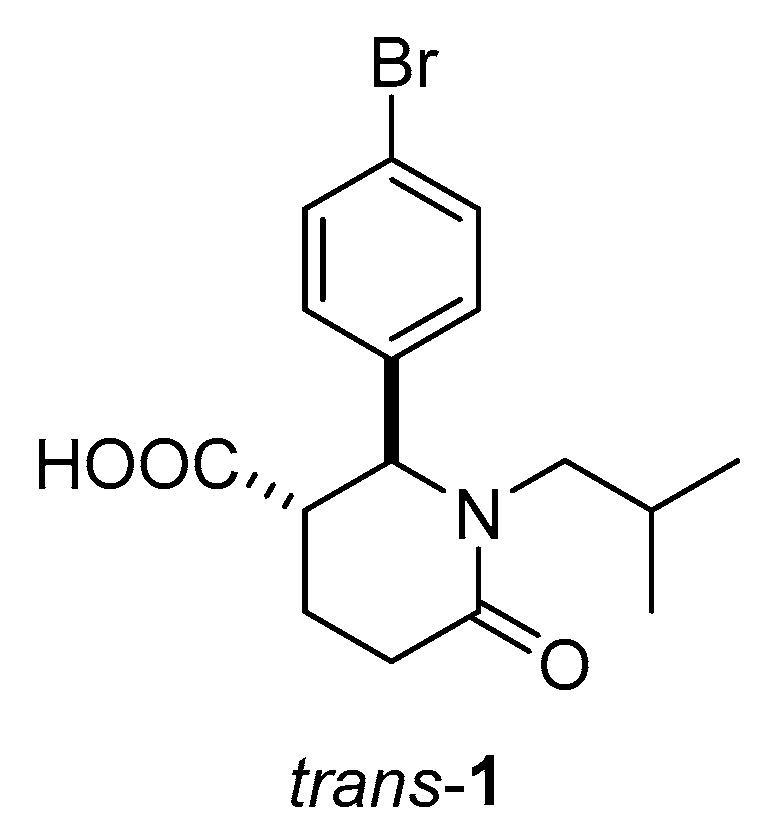
Structure of *trans*-2-(4-bromophenyl)-1-isobutyl-6-oxopiperidin-3-carboxylic acid (*trans*-**1**).

In this context, and in line with our ongoing studies, in the present work, we describe the synthesis, isolation and assignment of the absolute configuration (AC) of the enantiomers of *trans*-2-(4-bromophenyl)-1-isobutyl-6-oxopiperidin-3-carboxylic acid (*trans*-**1**) (Figure 3). This molecule can be considered the model compound of a wide range of aryl-carboxyl-substituted δ-lactams. Such structures are exceptionally important in Medicinal Chemistry, both as final products and as key intermediates for further modifications. Specifically, to provide a valuable method for the direct and complete resolution of *trans*-**1**, we exploited enantioselective High Performance Liquid Chromatography (HPLC). Moreover, to gain information on the enantiomer elution order, we used a HPLC-UV-ECD system (Electronic Circular Dichroism—ECD). A set of four different coated and immobilized cellulose- and amylose-based chiral stationary phases (CSPs) was investigated utilizing normal-phase and polar organic elution conditions. The effect of the mobile phase composition on enantioselectivity and retention was carefully evaluated. The best separation conditions were then properly scaled up to a (semi)preparative scale, thus obtaining enantiopure *trans*-**1**, which underwent X-ray diffraction analysis for the AC assignment. Actually, for chiral lactams, the assignment of the AC still represents a challenging issue. In fact, these intermediates are generally used as racemates for further derivatization and no robust and straightforward methodologies have been developed so far for their configurational study [42,43]. To this aim, thanks to the results of the present work, we provide a fully characterized δ-lactam, which represents an extremely useful reference compound for the AC assignment of structurally related lactams.

## 2. Results

### 2.1. Synthesis

**Scheme 2:**
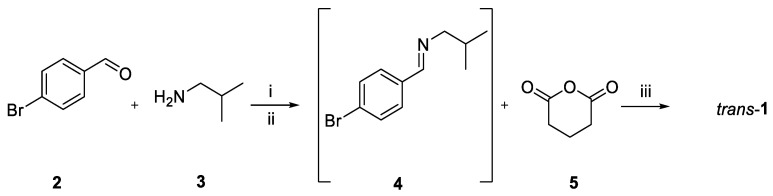
Reaction conditions: (i) 4 Å MS, Toluene, RT, 4 h; (ii) IRC_50_, Toluene, RT, 20 min.; (iii) *p-*xylene, reflux, 140 °C, 10 h.

δ-lactam **1** was synthesized via the CCR following the protocol already described by our team with suitable modifications (Scheme 2) [23]. Briefly, 4-bromobenzaldehyde and isobutylamine were reacted in toluene to generate imine-intermediate **4** in situ. The 4-bromobenzaldehyde group was chosen to facilitate the AC assignment by the X-ray diffraction analysis. To remove water and favor the formation of imine **4**, molecular sieves (4 Å, MS) were added [33]. Then, the carboxylic resin IRC50 was added to scavenge the unreacted isobutylamine, thus preventing the formation of side-products. Finally, the mixture was filtered and the solvent was evaporated. Intermediate **4** was then reacted with glutaric anhydride in *p*-xylene and the mixture was refluxed to form the desired δ-lactam. After liquid/liquid extraction, followed by flash chromatography, the final compound *trans*-**1** was successfully isolated with high purity (95%, *cis*/*trans* ratio: 10/90) and a satisfactory yield (38%).

### 2.2. Chiral Resolution

All the analyses have been performed using a HPLC-UV system coupled with a CD detector, thus obtaining on-line information on the elution order of the analytes. Four different CSPs were screened: two amylose-based CSPs, whose chiral selector are *tris-*(5-chloro-2-methylphenylcarbamate) coated on silica gel (Lux Amylose-2) and *tris*-(3,5-dimethylphenylcarbamate) immobilized on silica gel (Chiralpak IA), and two cellulose-based CSPs, containing *tris*-(3,5-dimethylphenylcarbamate) coated on silica gel (Chiralcel OD-H) and *tris*-(3,5-dichlorophenylcarbamate) immobilized on silica gel (Chiralpak IC) as chiral selectors. The elution has been performed using *n*-Hexane (*n*-Hex) combined with different concentrations of polar modifiers 2-propanol (IPA) or ethanol (EtOH) or using only alcohol. The mobile phases were added with trifluoroacetic acid (TFA, 0.3%) for both the coated CSPs and Chiralpak IC, or with a mixture of diethylamine (DEA, 0.1%) and TFA (0.3%) for Chiralpak IA. A standard screening protocol (see Supplementary Material, Table S2) was applied first, and then the elution conditions were properly modified to achieve a baseline separation of the analytes [44,45,46].

The conventional coated Lux Amylose-2 and Chiralcel OD-H CSPs allowed for poor or no peak separation, whereas good results in terms of peak separation, peak shape and enantioselectivity were obtained on immobilized Chiralpak IA and Chiralpak IC columns. Results are reported in Table 1 as retention factors (k1 and k2), separation factors (α) and resolution factors (Rs).

More in detail, good enantioresolution on Chiralpak IA was achieved by eluting with mixtures of alkane and polar cosolvent, while no peak separation was obtained in polar organic elution conditions. Specifically, baseline resolution was observed with *n*-Hex and a percentage of IPA or EtOH between 10 and 20% (for IPA, mobile phases A-C: α and Rs ranging from 1.53–1.56 and 2.20–4.28, respectively; for EtOH, mobile phases I-M: α and Rs ranging from 1.51–1.57 and 2.01–3.38, respectively, Table 1). Similarly, baseline separation of *trans*-**1** enantiomers was obtained on Chiralpak IC eluting with *n*-Hex and IPA, with an alcohol percentage between 10 and 30% (mobile phases A-E: α and Rs ranging from 1.30–1.45 and 1.48–3.91, respectively, Table 1). Marginal or null enantioseparation was observed eluting with *n*-Hex and EtOH in all the mobile phase compositions experimented on. Again, no peak separation was obtained eluting with pure alcohol.

**Figure 4:**
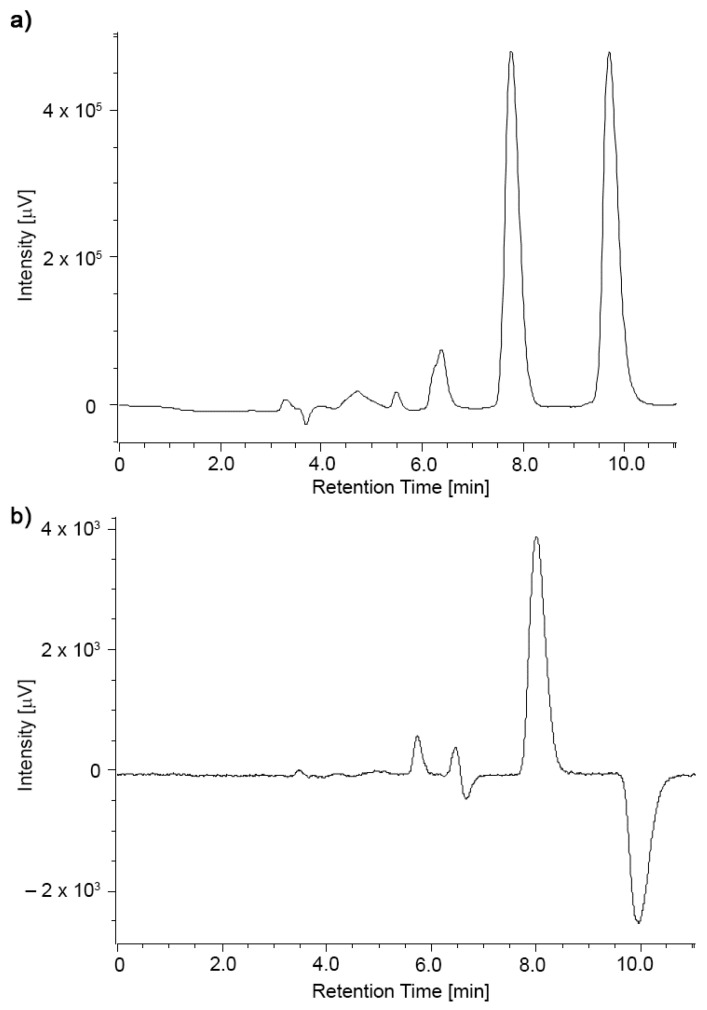
Analytic separation of *trans-***1** on Chiralpak IA; eluent: n-Hex/IPA/DEA/TFA (90:10:0.1:0.3, *v*/*v*/*v*/*v*), flow rate: 1 mL/min, (**a**) UV trace (detection at 220 nm), (**b**) ECD trace (detection at 240 nm).

**Figure 5:**
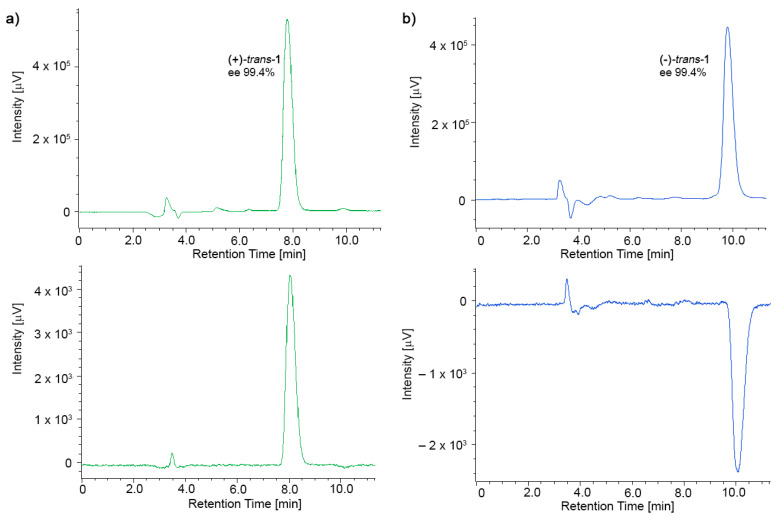
UV trace (up) and ECD trace (down) of the first eluted (+)-*trans*-**1** (**a**) and the second eluted (−)-*trans*-**1** (**b**) using Chiralpak IA, mobile phase M (Table 1).

A good enantioselectivity (α = 1.51; RS = 3.38; Table 1) was observed in short retention times (t_r1_ = 7.7 min; t_r2_ = 9.7 min) (Figure 4), using the Chiralpak IA column and eluting with *n*-Hex/EtOH/DEA/TFA (90:10:0.1:0.3, *v*/*v*/*v*/*v*). Moreover, the first eluted enantiomer showed a positive peak, whereas the second eluted enantiomer had a negative peak of the same intensity. The broad peak at t_r_ = 6.4 min of the UV Chromatogram corresponds to two peaks with opposite Cotton effects in the ECD trace, suggesting that the analyte is an almost unresolved racemic mixture. The peak area is about 10%, which is consistent with the amount of *cis* isomers typically obtained as byproduct during the CCR. Considering the good performance of this chromatographic method, these experimental conditions were properly scaled-up to the (semi)-preparative scale. Resolution of *trans*-**1** in (semi)-preparative scale was accomplished using a Chiralpak IA column according to conditions summarized in Table 2. Fractions collected (see Supplementary Material, Figure S3) were analyzed using the previously identified analytical method (Chiralpak IA, mobile phase M, Table 1). Actually, 50 mg of *trans*-**1** were processed. After 17 cycles, 18.1 mg of the first eluted enantiomer ([${\left[\alpha \right]}_{D}^{20}$ = +4.8, c ≅ 0.5%, CHCl_3_) and 21.2 mg of the second eluted enantiomer ((${\left[\alpha \right]}_{D}^{20}$ = −4.8 c ≅ 0.5%, CHCl_3_) were isolated with *e.e.* higher than 99% (Figure 5), together with 8.5 mg of an intermediate fraction as a mixture of the two enantiomers.

### 2.3. Single Crystal X-ray Diffraction Study

To obtain single crystals suitable for the X-ray diffraction, several attempts were performed on both (+)-*trans*-**1** and (−)-*trans-***1** varying solvents and crystallization conditions. The first eluted enantiomer, (+)-*trans*-**1**, was successfully crystallized at room temperature from IPA in a water vapor saturated environment, and the crystal obtained underwent the X-ray diffraction analysis (crystal data are reported in the Supplementary Material, Table S3.).

**Figure 6:**
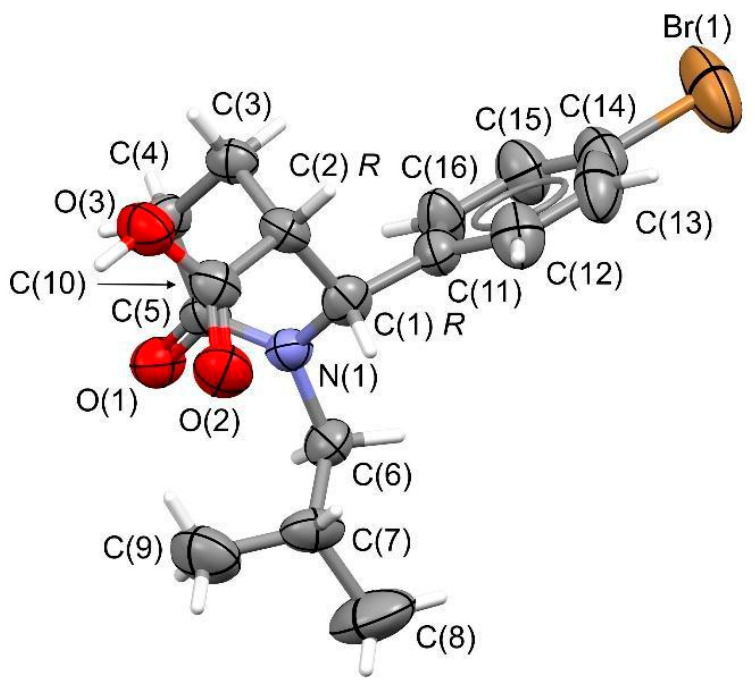
Molecular structure (drawn as plot showing thermal ellipsoids at the 50% probability level) by X-ray crystallography of first eluted (+)-(2*R*,3*R*)-**1**.

The molecular structure revealed by the X-ray diffraction study is reported in Figure 6. The first eluted, (+)-*trans*-**1**, exhibits a slightly distorted half-chair conformation of the six-membered ring defining the δ-lactam, with five atoms almost coplanar (maximum deviation of 0.058 (5) Å from their best plane) and the bridgehead C atom carrying the carboxyl out of this plane by 0.680 (9) Å. The two chiral C atoms bind the carboxyl and aromatic substituents according to a *trans* configuration. The absolute configuration *R* for both chiral centers of the six membered ring was assessed on the basis of the analysis of the anomalous scattering effects for the measured reflections. The final calculated Flack x parameters of −0.03 (1) confirms that the absolute structure was correctly assigned and that only the enantiomer shown in Figure 6 occurs in the crystal.

**Figure 7:**
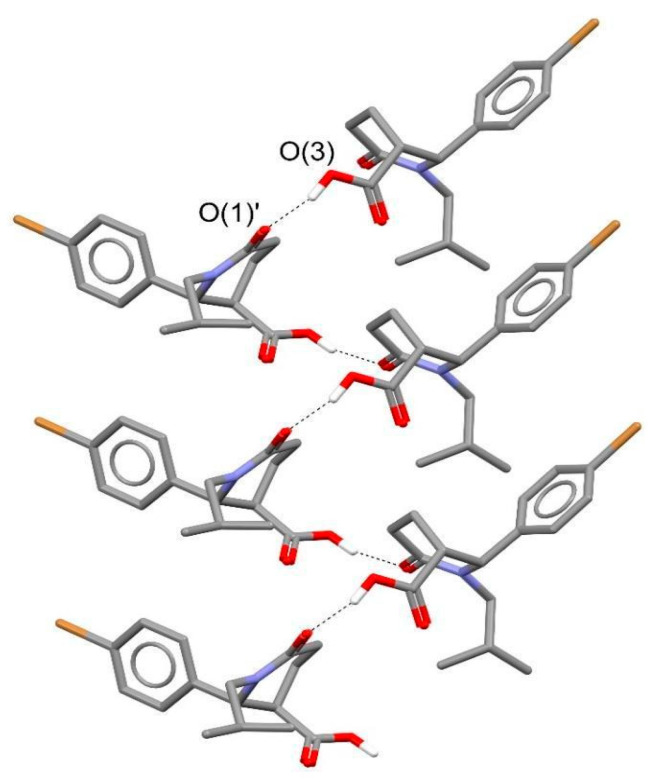
A simplified sketch showing the symmetrically equivalent intermolecular O-H···O hydrogen bonds (dashed lines) occurring in the crystal of the (+)-(2*R*,3*R*)-**1** compound (atom names are reported only for O atoms involved in the H-bond, hydrogens not involved in the H-bonds are omitted for clarity).

At the solid state, adjacent molecules are linked together by means of O-H···O hydrogen bonds, where the OH of the carboxylic group acts as H-donor and the O atom of the carbonyl group is the H-acceptor. The geometrical features of the O-H···O interaction are: O(3) ···O(1)’ 2.613(7) Å, H(3O) ···O(1)’ 1.74(3) Å, O(3)-H(3O)···O(1)’ 163(8)° (symmetry code: (‘) = ^1^/_2_ + *x*, ^1^/_2_ − *y*, 1 − *z*), and these interactions originate a supramolecular double chain of (+)-(2*R*,3*R*)-**1** molecules extending along the direction of the a crystallographic axis (Figure 7). The supramolecular synthon [47] originating from this H-bond motif can be described with the graph set notation C(8) [48]. Inspection of the available structural data in the literature [49] shows that such a synthon occurs in different crystal structures of several δ-lactam derivatives having the carboxylic group at the 3-position of the ring [32,50,51,52]. To sum up, the absolute configuration of the first eluted (+)-*trans*-**1** was elucidated through X-ray diffraction experiments and both chiral C centers resulted *R*-configured.

## 3. Discussion

**Scheme 3:**
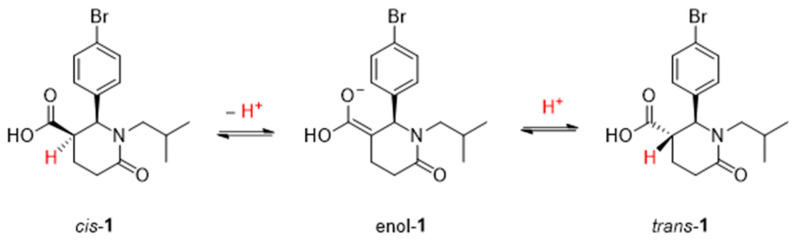
Rationalization of the observed trans-selectivity in the CCR conducted under thermodynamic control. The enol form which is obtained upon deprotonation (loss of the red hydrogen in the scheme) will lead to the most stable product under reversible conditions.

δ-lactam **1** was synthesized via the CCR following procedure outlined in Scheme 2. Consistent with literature data, the reaction proceeded with high diastereoselectivity, predominantly yielding the *trans* isomer **1** (*cis*/*trans* ratio of 10/90, NMR analysis, see Supplementary Material Figure S1 and Table S1), as confirmed by NOE experiments (see Supplementary Material Figure S2) [29,30]. The observed stereoselectivity is due to the fact that the reaction is performed under thermodynamic control (*p*-xylene, reflux, 140 °C, 10 h) and, therefore, the main product will be the most thermodynamically stable. Since the *trans* product has less steric collision between the aromatic group and the carboxylic acid, it is formed mainly under reversible conditions. As outlined in Scheme 3, the position of the stereocenter attached to the carboxyl group is enolizable and this explains the *cis*/*trans* equilibrium in which the *trans* product predominates. Furthermore, the obtained *cis*/*trans* ratio is in accordance with the analysis reported in Figure 4, in which the UV chromatogram shows a broad peak at t_r_ = 6.4 min with 10% of peak area attributable to racemic *cis*-**1** (two peaks with opposite sign are present in the ECD trace). Overall, the synthetic protocol afforded quick, easy and efficient access to the desired product.

To isolate the enantiomers of *trans*-**1,** we exploited enantioselective HPLC resolution on polysaccharide-based CSPs, as a valuable strategy for obtaining both enantiomers with high enantiomeric purity and yield [53,54,55]. Because such CSPs exhibit enantioselectivity in several mobile phase types, we applied the screening protocol reported in the Supplementary Material, Table S2 [44,45]. Specifically, we exploited amylose- and cellulose-based CSPs both in a normal-phase mode (NPM), eluting with a mixtures of *n*-hexane (nonpolar hydrocarbon solvent) and in alcohol of a low molecular weight (IPA and EtOH), and in a polar-organic mode. Pure EtOH was used as an intermediate solvent in between the normal-phase mode and the polar organic mode. Only when the results of the screening were encouraging, but not fully successful, did we modify the mobile phase to achieve a compound baseline separation. An ECD spectrometer coupled with HPLC-UV was used as a chiroptical detector, since it can allow the determination of the elution order of enantiomers in a racemic mixture at the analytical stage.

Enantioseparation of *trans*-**1** was obtained on immobilized Chiralpak IA and Chiralpak IC, in NPM elution conditions. The first eluted enantiomer shows positive peak in the ECD trace, whereas the second eluted isomer shows a negative peak in both the columns, eluting with mobile phases characterized by a high percentage of *n*-hexane and a low percentage of alcohol (IPA or EtOH for Chiralpak IA and IPA for Chiralpak IC). Thus, despite the different polysaccharide nature of the CSPs, amylose and cellulose-based, in both immobilized columns, the enantiomers preserve the same elution order.

The results obtained on an analytical scale were fully considered in the perspective of scaling-up the enantioseparation to a (semi)-preparative level to isolate enantiomeric *trans*-**1** in multi-milligram quantities. To reach an economic and efficient preparative enantiomer separation, many important requirements must be satisfied, such as the shortest retention times, high solubility of racemate and enantiomers in the eluent/injection solvent and the use of a mobile phase consisting of a pure low-cost solvent to facilitate workup and re-use of the mobile phase [56]. Thus, after solubility studies employing different mixtures of *n*-Hex and alcohol, elution condition M (Table 1) was chosen for (semi)-preparative scales. Chiralpak IA, eluting with *n*-Hex 90/EtOH 10 added with TFA and DEA, was selected for scaling-up purposes, showing a similar enantioresolution and equal baseline separation to the other best conditions reported here before. A 25 cm column was used at a flow rate of 2.5 mL/min with 3 mg injected at each cycle. Racemic *trans*-**1** (50 mg) was processed and enantiomers delivered with an enantiomeric excess higher than 99% and a yield of 74% and 82%, for the first and the second eluted enantiomers, respectively.

To determine the absolute configuration for individual enantiomers of *trans*-**1**, we performed a single-crystal X-ray diffraction analysis. In fact, the presence of heavy atoms such as bromine allows to improve the resonant scattering properties, thus facilitating the determination of the AC by X-ray diffraction [57]. The single crystals for X-ray experiments were grown from samples by vapor diffusion, using IPA as solvent and water as precipitant. The enantiomer (+)-*trans*-1 yielded a single crystal suitable for X-ray diffraction. The analysis of the ORTEP diagram and the Flack x parameters [58] of −0.03(1), which also indicates that the whole crystal is homochiral, and revealed that the AC of (+)-*trans*-**1** is 2*R*,3*R*.

The assignment of AC of *trans*-**1** provides a viable reference compound for determining the AC of other structurally related lactams, since compounds with a similar chemical environment close to the stereocenter will display comparable profiles of both ECD and VCD spectra. Specifically, enantiomeric *trans*-**1** may be considered the model compound of a wide range of aryl-carboxyl-substituted δ-lactams.

## 4. Materials and Methods

### 4.1. General

Solvent evaporation was carried out under reduced pressure by a Heidolph Laborota 4000 instrument (Heidolph Instruments GmbH & Co., Schwabach, Germany). Analytical thin-layer chromatography (TLC) was performed on silica gel precoated with aluminium-backed plates (Fluka Kieselgel 60 F254, Merck, Darmstadt, Germany). An UV light (λ = 254 nm) was used for the detection. Flash chromatography was performed with silica gel 60 (particle size 230–400 mesh) purchased from Nova Chimica (Cinisello Balsamo, Italy). All the reactants and deuterated solvent were supplied by Sigma Aldrich (Milan, Italy). HPLC grade-solvents were purchased from Honeywell (Seelze, Germany), and analytical grade-solvents from PanReac (Darmstadt, Germany).

Optical rotation values were measured on the Jasco photoelectric polarimeter DIP 1000 (Tokyo, Japan) with a 0.5 dm quartz cell at the sodium D line (λ = 589 nm); the compound was dissolved in chloroform in a concentration of 0.5% (*w*/*v*).

NMR experiments were carried out at 298 K on a Bruker Avance III 400 MHz spectrometer (Milan, Italy). The NMR experiments were in 500 μL of CDCl_3_.

The purity of the final compound was assessed via HPLC analysis on a Jasco system (Jasco, Tokyo, Japan) consisting of a PU-1580 pump, 851-AS, autosampler, MD-1510 Photo Diode Array (PDA) detector using a Phenomenex Synergi 4u Hydro-RP 80A (50 × 2 mm, 4 μm) under the following conditions: flux: 1 mL/min, detection at λ = 254 nm, eluent: from H_2_O:ACN 90:10 + 0.1% of HCOONH_4_ to H_2_O:ACN 10:90 + 0.1% of HCOONH_4_ over 9 min.

### 4.2. Synthesis of 2-(4-Bromophenyl)-1-isobutyl-6-oxopiperidin-3-carboxylic Acid (trans-***1***)

In a round-bottom flask, 4-bromobenzaldehyde (100 mg, 0.54 mmol) and isobuthylamine (53.7 μL, 0.54 mmol) were dissolved in toluene (2 mL). To remove water from the reaction environment, 4 Å molecular sieves (MS) (65 mg) were added and the reaction mixture was stirred at room temperature for 4 h. MS were then removed by filtration and the amberlyst carboxylic resin IRC50 (54 mg, 10 meq/g) was added. The mixture was kept under mechanical stirring for 20 min and then, after IRC50 filtration, the solvent was evaporated under reduced pressure. The yellowish oil obtained was dissolved in *p*-xylene (2 mL) and glutaric anhydride (61.6 mg, 0.54 mol) was added. The reaction mixture was stirred at 140 °C for 10 h and subsequently the yellow solution was evaporated in vacuo. The residue was dissolved in ethyl acetate (10 mL) and extracted with 5% NaHCO_3_ (20 mL). The water phase was added with 10% HCl (pH ≈ 3.0) and extracted with dichloromethane (DCM) (30 mL). The combined organic layers were dried over Na_2_SO_4_ and evaporated in vacuo, furnishing the crude product as a yellow oil. The crude was finally purified by flash chromatography eluting with DCM/MeOH (9:1, *v*/*v*), yielding racemic *trans*-**1** as a yellow oil (72.7 mg, 38%).

^1^H-NMR (400 MHz, CDCl_3_, δ): 7.51 (m, Ar 2H), 7.08 (m, Ar 2H), 5.10 (m, 1H), 3.96–2.17 (m, 2H; CH_2_), 2.84 (m, 1H), 2.71–2.55(m, 2H; CH_2_), 2.09–1.90 (m, 2H; CH_2_), 2.01 (m, 1H), 0.87 (s, 6H, CH_3_).

^13^C-NMR (100 MHz, CDCl_3_, δ): 132.2 (Ar 2C), 128.4 (Ar 2C), 61.4, 52.6 (2C), 46.2, 29.5 (2C), 26.4, 18.9 (2C), 14.2 (2C, CH_3_).

### 4.3. Chiral Chromatographic Resolution of Trans-***1***

The HPLC runs were performed on a Jasco system (Japan) consisting of a PU-1580 pump, 851-AS, autosampler, MD-1510 Photo Diode Array (PDA) detector and Electronic Circular Dichroism (ECD) 2095 Plus detector.

For the analytical screening, Chiralpak^TM^ IC (0.46 cm diameter × 25 cm length, 5 µm), Chiralcel^TM^ OD-H (0.46 cm diameter × 15 cm length, 5 µm), Chiralpak^TM^ IA (0.46 cm diameter × 25 cm length, 5 µm) (all produced by Daicel Industries Ltd., Tokyo, Japan) and Lux 5u Amylose-2^TM^ (0.46 cm diameter × 15 cm length, 5 µm) (produced by Phenomenex, Torrance, CA, USA) were used as CSPs. Different compositions of *n*-Hex-alcohol (IPA or EtOH) mixture or pure alcohol were used as mobile phase and DEA (0.1%) and TFA (0.3%) were added to mobile phase for analysis on immobilized Chiralpak^TM^ IA column, while only TFA (0.3%) was added for analysis on coated Chiralcel^TM^ OD-H and Lux 5u Amylose-2^TM^ columns as well as for analysis on Chiralpak^TM^ IC. *trans*-**1** was dissolved in IPA and analyzed at room temperature under the following conditions: flow rate 1 mL/min (unless otherwise specified), UV detection at 220, ECD detection at 240 nm and injection volume 10 µL.

Chromatogram acquisitions and elaborations were carried out by the ChromNAV software. It directly furnished the resolution factor, while retention factors (k1 and k2) of the two enantiomers and selectivity (α) were calculated according to Equation (1) and Equation (2), respectively.

(1) $$k=\frac{t_{r}-\;t_{0}}{t_{0}}$$

with *t_r_* = retention time, and *t*_0_ = solvent front

(2) $$\alpha =\;\frac{k_{2}}{k_{1}}$$

For the (semi)-preparative runs, the Chiralpak^TM^ IA column (1 cm × 25 cm length, 5 μm) was used eluting with n-Hex/EtOH/DEA/TFA acid (90:10:0.1:0.3, *v*/*v*/*v*/*v*) at a flow rate of 2.5 mL/min, UV detection was at 220 nm. *trans-***1** was dissolved in the mobile phase (c ≅ 3mg/mL) and the injection volume was 1 mL.

### 4.4. X-ray Analysis

A concentrated solution of each pure enantiomer of **1** in IPA was prepared in a small vial. The vials containing the enantiomers were put into a larger vessel containing the anti-solvent, i.e., distilled water. The larger vial was sealed to allow the crystal growth.

Diffraction data for a colorless crystal of the (+)-*trans*-**1** were collected at ambient temperature by means of a Bruker-AXS three circle diffractometer (Bruker AXS Inc., Madison, WI, USA) working with graphite monochromated Mo-Kα X-radiation (λ = 0.7107 Å) and equipped with the SMART-APEX bidimensional CCD detector.

Data reduction (including intensity integration, background, Lorentz and polarization corrections) was performed with the SAINT software [59]. Absorption effects were empirically evaluated by the SADABS software and absorption correction was applied to the data [60].

Crystal structure was solved by direct methods (SIR 97) [61] and refined by full-matrix least-square procedures on *F*^2^ using all reflections (SHELXL-2018) [62]. Anisotropic displacement parameters were refined for all non-hydrogen atoms. Hydrogens bonded to C atoms were placed at calculated positions with the appropriate AFIX instructions and refined using a riding model; hydrogen bonded to an O atom was located in the final ∆*F* maps; its position was successively refined during the final least-square procedures restraining the O-H distance to be 0.90 ± 0.01 Å. CCDC 2,031,039 contains the supplementary crystallographic data for the studied compound. These data can be obtained free of charge from The Cambridge Crystallographic Data Center via www.ccdc.cam.ac.uk/data_request/cif.

## 5. Conclusions

In the last few years, the research on γ- and δ-lactam scaffolds for the discovery of new biologically active molecules has grown consistently. Considering that most of those molecules show at least one chiral center, and keeping in mind that the enantiomers of a chiral compound may interact differently with biological systems, a key step in the drug discovery process is the investigation of the role of chirality in the biological activity of lactam-based molecules. Since no robust and straightforward methodologies for the configurational study of chiral lactams have been developed so far, the assignment of the absolute configuration of the molecules still represents a challenging task. To develop a fast and efficient methodology for the configurational study of molecules characterized by a chiral lactam scaffold, in the present work, we prepared enantiopure *trans*-**1** via chiral HPLC, and assigned the absolute configuration to the enantiomers by an X-ray diffraction method using the Flack parameter. The absolute structure of (+)-*trans*-**1** crystal and, consequently, the absolute configuration of its chiral constituents, (+)-(2*R*,3*R*)-**1** molecules, was established by us on this basis. This compound represents a valuable reference compound for the configurational study of structurally related lactams.

## Figures and Tables

**Table 1:** ^a^ Eluent composition: A, *n*-Hex/IPA (90:10 *v*/*v*); B, *n*-Hex/IPA (85:15 *v*/*v*); C, *n*-Hex/IPA (80:20 *v*/*v*); D, *n*-Hex/IPA (75:25 *v*/*v*); E, *n*-Hex/IPA (70:30 *v*/*v*); F, *n*-Hex/IPA (50:50 *v*/*v*); G, IPA (100); H, EtOH (100); I, *n*-Hex/EtOH (80:20 *v*/*v*); L, *n*-Hex/EtOH (85:15 *v*/*v*); M, *n*-Hex/EtOH (90:10 *v*/*v*); ^b^ Mobile phase was added with 0.1% DEA; ^c^ Mobile phase was added with 0.3% TFA; Flow rate: 1.0 mL/min; ^d^ Flow rate: 0.5 mL/min; Concentration: 1 mg/mL; Injection volume: 10 μL; UV detection at 220 nm.

CSPs								
Chiralpak IA ^b,c^					Chiralpak IC ^c^			
Eluent ^a^	k1	k2	α	RS	k1	k2	α	RS
**A**	1.39	2.17	1.56	4.28	4.15	5.47	1.32	3.91
**B**	0.74	1.15	1.56	3.19	0.94	1.37	1.45	2.99
**C**	0.49	0.75	1.53	2.20	1.44	1.87	1.30	2.33
**D**	0.53	0.80	1.51	1.59	1.16	1.54	1.33	1.75
**E**	0.32	0.47	1.44	1.01	0.93	1.22	1.31	1.48
**F**	0.19	0.28	1.46	0.81	0.36	0.49	1.35	-
**G ^d^**	0.24	-	-	-	0.47	-	-	-
**H ^d^**	0.24	-	-	-	0.48	-	-	-
**I**	0.65	0.99	1.53	2.01	0.68	0.78	1.14	-
**L**	0.86	1.36	1.57	2.67	0.94	1.08	1.15	-
**M**	1.43	2.16	1.51	3.38	1.93	2.20	1.14	0.99

**Table 2:** (Semi)-preparative HPLC parameters for separating the enantiomers of *trans-***1.**

(2*R*,3*R*/2*S*,3*S*)-1				
CSP	Eluent (*v*/*v*/*v*/*v*)	Flow Rate	Injection Volume	Concentration
Chiralpak IA (1 cm × 25 cm, 5 μm)	*n*-Hex/EtOH/TFA/DEA 90:10:0.1:0.3	2.5 mL/min	1 mL	3 mg/mL

## Supplementary Materials

The following are available online at, Figure S1: **^1^**H-NMR spectra of *trans*-**1**, Figure S2: NOE bidimensional spectra of *trans*-**1**, Figure S3: (Semi)-preparative enantiomer separation of *trans-***1** on Chiralpak IA^TM^, Table S1: ^1^H-NMR and ^13^C-NMR signals of *trans*-**1**, Table S2: Mobile phase composition of the standard screening protocol applied in analytical scale, Table S3: Crystal data of (+)-*trans*-**1.**
