# Supplementary material for: Enantiomeric Resolution and Absolute Configuration of a Chiral δ-Lactam, Useful Intermediate for the Synthesis of Bioactive Compounds
Source: Molecules. 2020 Dec 19;25(24):6023. doi: 10.3390/molecules25246023 (PMC7766352; doi:10.3390/molecules25246023)
Supplement: Supplementary file 1 [file molecules-25-06023-s001.pdf]

Supplementary material

S.1 NMR analysis of *trans*-1

^{1}H-NMR spectra of *trans*-**1** is reported in Figure S.1.; ^{1}H-NMR and ^{13}C-NMR signals of *trans*-**1** are reported in table S.1; the NOESY spectra is stated in Figure S.2. As expected, no NOE effect between the hydrogen atoms on the chiral centers C2 and C3 was observed.

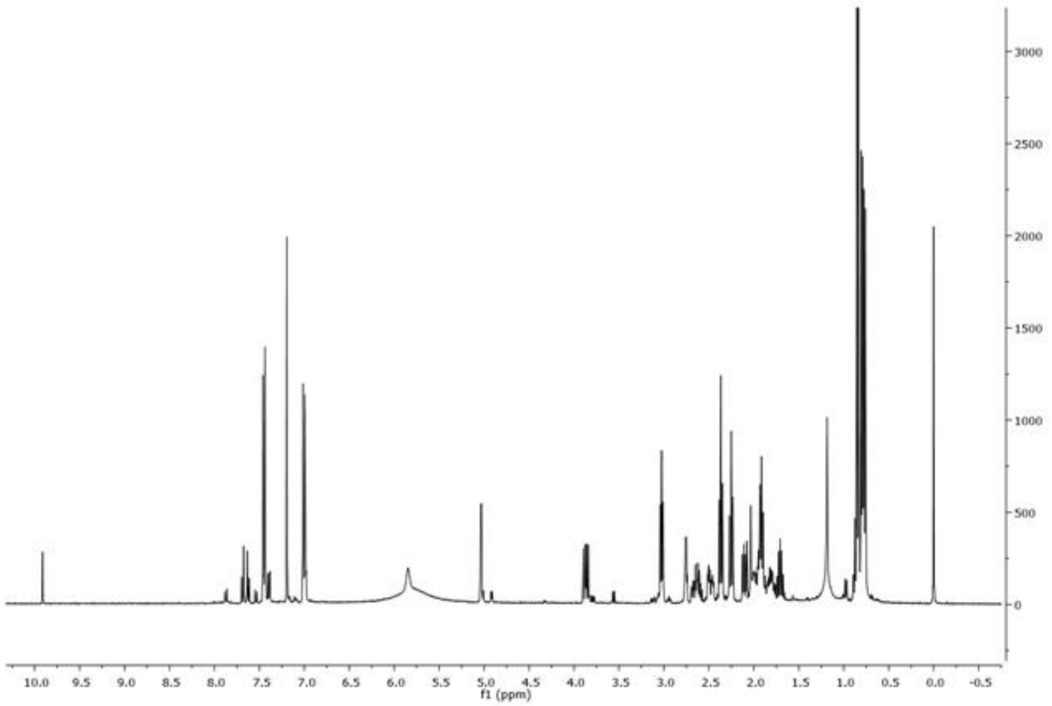

Figure S.1^{1}H- NMR spectra of *trans*-**1**.

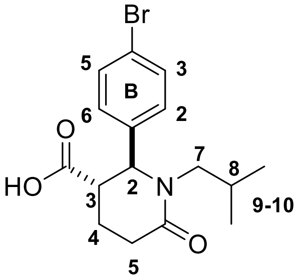

trans-**1**

	^{1}H-NMR (δ ppm)	^{13}C-NMR (δ ppm)
**B3,5**	7.51	132.2
**B2,6**	7.08	128.4
**2**	5.10	61.4
**7a**	3.96	52.6
**3**	2.84	46.2
**5a**	2.71	29.5
**5b**	2.55	29.5
**7b**	2.17	52.6
**4a**	2.09	18.9
**8**	2.01	26.4
**4b**	1.90	18.9
**9-10**	0.87	14.2

Table S.1. ^{1}H-NMR and ^{13}C-NMR signals.

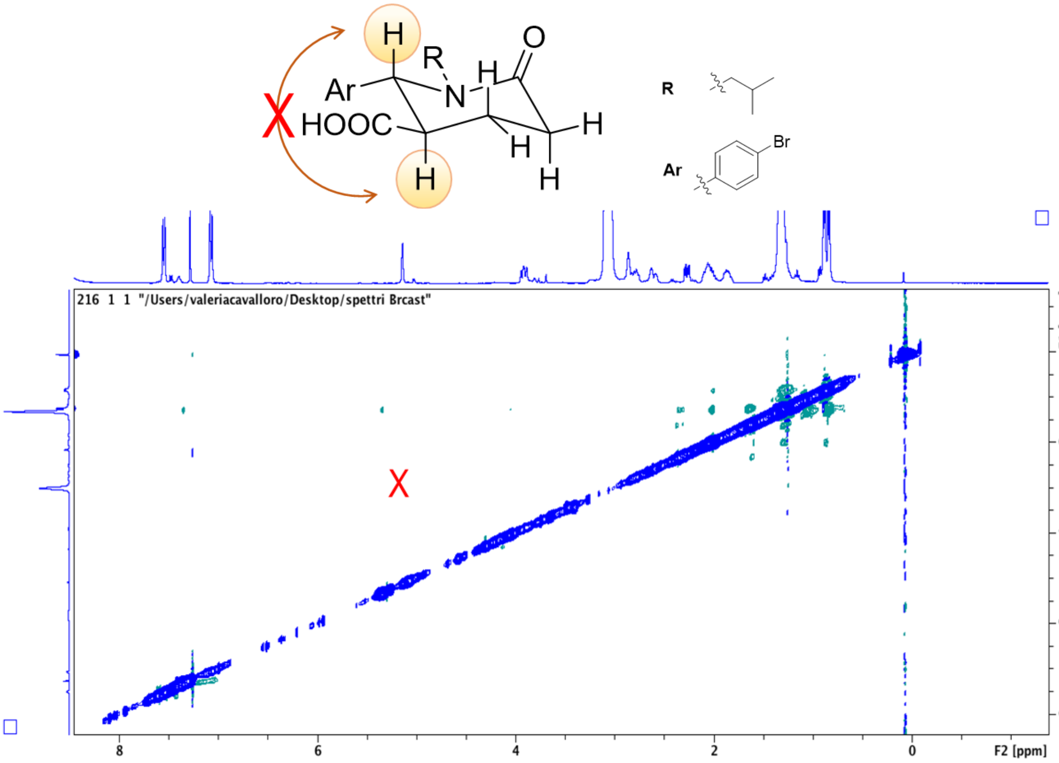

Figure S.2 NOE bidimensional spectra of *trans*-**1**.

S.2 Standard screening protocol of mobile phase

The standard screening protocol of mobile phase composition used for the first studies on enantioseparation of *trans*-**1** is reported in Table S.2. [1,2]

Mobile Phase Composition			
Entry	n-Hexane	2-propanol	Ethanol
1	90	10	
2	50	50	
3		100	
4			100
5	90		10

Table S.2. Standard screening protocol: mobile phase composition.

S.3 (Semi)-preparative enantioseparation of *trans*-**1** via chiral HPLC

The chromatogram of a (semi) preparative run is reported in Figure S.3 as an example. Fractions were collected according to dashes.

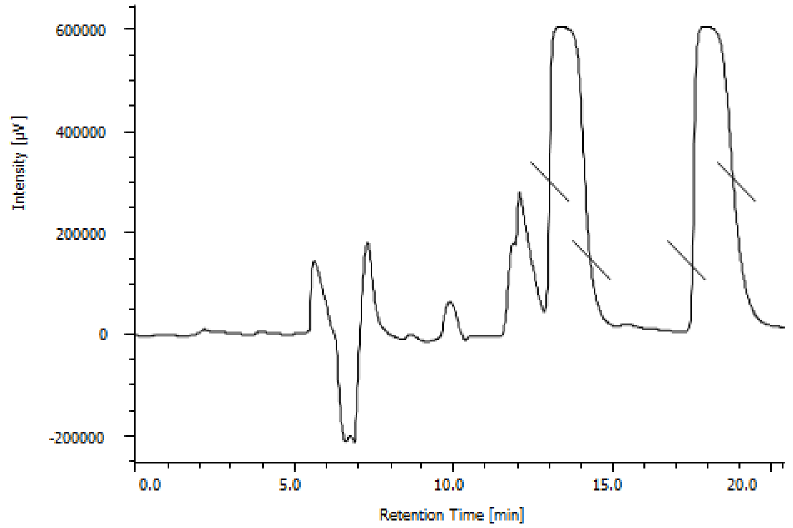

Figure S.3 (Semi)-preparative enantiomer separation of *trans*-**1** on Chiralpak IA (1 cm × 25 cm, 5 µm) eluting with n-Hex/ EtOH/DEA/TFA (90:10:0.1:0.3, v/v/v/v), flow rate 2.5 ml/min. UV detection at 220 nm. Cut points given by dashes (—).

S.4 Single crystal x-ray diffraction study

Crystal data obtained for *(+)-trans*-**1** by X-ray diffraction analysis are reported in table S.3.

Formula	$C_{16}H_{20}BrNO_3$	Scan type	*ω* scans
M	354.23	θ range (°)	1.87 - 25.06
Dimension (mm)	0.22 x 0.06 x 0.05	Measured reflections	14540
Crystal system	orthorhombic	Unique reflections	2868
Space group	$P\ 2_12_12_1$ (no. 19)	*R*int	0.0062
a (Å)	6.8032(7)	Strong data [$I_O>2\sigma(I_O)$]	1953
b (Å)	14.5352(16)	Refined parameters	193
c (Å)	16.4164(18)	*R*1, w*R*2 strong data	0.0527, 0.1160
V (Å^3)	1623.4(3)	*R*1 w*R*2 all data	0.0891, 0.1376
Z	4	GoF	1.020
ρ_{calcd} (g cm^{-3})	1.449	Flack x parameter [3]	-0.025(8)
µ Mo-Kα [mm^{-1}]	2.541	max/min residuals (eÅ^{-3})	0.62/-0.67
min/max trans. factors	0.746/0.862		

Table S.3. Crystal data of (+)-*trans*-**1.**

Reference

1. Cavalloro, V.; Russo, K.; Vasile, F.; Pignataro, L.; Torretta, A.; Donini, S.; Semrau, M.S.; Storici, P.; Rossi, D.; Rapetti, F.; et al. Insight into GEBR-32a: Chiral Resolution, Absolute Configuration and Enantiopreference in PDE4D Inhibition. *Mol. Basel Switz.* **2020**, 25, doi:10.3390/molecules25040935.

2. Rossi, D.; Pedrali, A.; Marra, A.; Pignataro, L.; Schepmann, D.; Wünsch, B.; Ye, L.; Leuner, K.; Peviani, M.; Curti, D.; et al. Studies on the enantiomers of RC-33 as neuroprotective agents: isolation, configurational assignment, and preliminary biological profile. *Chirality* **2013**, 25, 814–822, doi:10.1002/chir.22223.

3. Parsons, S.; Wagner, T. Use of intensity quotients and differences in absolute structure refinement. *Acta Crystallogr.* **2013**; *B69*: 249-259, doi:10.1107/S2052519213010014.
